# The Effects of Foot Reflexology on Chemotherapy-Induced Nausea and Vomiting in Patients with Digestive System or Lung Cancer: Protocol for a Randomized Controlled Trial

**DOI:** 10.2196/17232

**Published:** 2020-07-14

**Authors:** Audrey Murat-Ringot, Pierre Jean Souquet, Marion Chauvenet, Charlotte Rentler, Fabien Subtil, Anne-Marie Schott, Marie Preau, Vincent Piriou

**Affiliations:** 1 Centre de Coordination en Cancérologie Hospices Civils de Lyon Pierre-Benite France; 2 HESPER EA7425 Université Lyon 1 Lyon France; 3 Laboratoire GREPS EA 4163 Institut de Psychologie Université Lyon 2 Bron France; 4 Laboratoire de Biométrie et Biologie Evolutive UMR 5558 Centre National de la Recherche Scientifique Université Lyon 1 Villeurbanne France; 5 Pôle Santé Publique Hospices Civils de Lyon Lyon France; 6 Réanimation Anesthésie Hospices Civils de Lyon Pierre-Benite France

**Keywords:** cancer, randomized controlled trial, foot reflexology, nausea, vomiting, chemotherapy

## Abstract

**Background:**

The side effects of chemotherapy, specifically chemotherapy-induced nausea and vomiting, are a concern for patients. To relieve these side effects, antiemetic drugs are recommended. However, some patients report that these drugs are not sufficiently effective. Moreover, patients with chronic disease, including cancer, are increasingly interested in complementary and alternative medicines, and express the desire for nonpharmacological treatments to be used in hospitals. Foot reflexology is a holistic approach that is reported to significantly reduce the severity of chemotherapy-induced nausea and vomiting in patients with breast cancer. Some of the chemotherapy treatments for patients with lung and digestive system cancer are moderately or highly emetic.

**Objective:**

The primary objective of this study is to assess the benefits of foot reflexology, together with conventional treatments, on the severity and frequency of chemotherapy-induced nausea and vomiting in patients with lung or digestive system cancer. The secondary objectives to be assessed are quality of life, anxiety, and self-esteem.

**Methods:**

This study is an open-label randomized controlled trial conducted over 22 months (18 months intervention and 4 months follow-up). Eligible participants are patients with a lung or digestive system cancer with an indication for platinum-based chemotherapy. Participants are randomized into two groups: conventional care with foot reflexology and conventional care without foot reflexology. Foot reflexology sessions (30 minutes) are performed on an outpatient or inpatient basis. It was estimated that 40 participants per group will be required. The benefits of foot reflexology will be assessed by comparing the relative change in the severity of nausea and vomiting, as assessed by a visual analogue scale, and the frequency of these side effects between the two groups. The secondary objectives will be assessed with the European Organization for Research and Treatment of Cancer Quality of Life Questionnaire; Hospital and Anxiety Depression Scale; and Body Image Questionnaire.

**Results:**

This study was approved by the regional ethics committee (Île de France X CPP) on April 3, 2018 (No. ID RCB 2018-A00571-54). Enrollment started in June 2018. Data analysis will be performed during the second quarter of 2020 and results will be published in the last quarter of 2020.

**Conclusions:**

The lack of knowledge regarding the efficacy and safety of foot reflexology limits oncologists to recommend it for this use. This study will provide evidence of the benefits of foot reflexology. If efficacy is confirmed, foot reflexology may be a promising complement to conventional antiemetic drugs.

**Trial Registration:**

Clinicaltrials.gov NCT03508180; https://www.clinicaltrials.gov/ct2/show/NCT03508180.

**International Registered Report Identifier (IRRID):**

DERR1-10.2196/17232

## Introduction

### Background

Patients with cancer are increasingly interested in complementary and alternative medicines (CAMs) [1]. According to the systematic review by Keen et al [2], the main reasons patients use CAMs are to treat their cancer, to treat the side effects of treatment, and to improve their quality of life. Patients with chronic disease, including cancer, express the desire for nonpharmacological treatments and CAMs be used in hospitals [3].

At the time of writing, the most frequently provided CAMs in private and public oncology centers in European countries are mind-body techniques, acupuncture, homeopathy, energy therapies, health promotion, traditional herbal medicine, as well as manipulation and body-based practices (kinesiology, osteopathy, physiotherapy, and reflexology) [4]. Foot reflexology is a holistic approach. A systematic review indicated that foot reflexology seems to be effective for patients with cancer but this field requires further rigorous research with a randomized controlled trial [5]. More specifically, foot reflexology improved the quality of life of patients in the palliative stage of cancer [6], significantly decreased pain intensity and anxiety in patients with metastatic cancer [7], and significantly decreased the perceived pain and anxiety of postoperative patients with gastric cancer and hepatocellular cancer [8]. Moreover, a significant decrease in chemotherapy-induced nausea and vomiting (CINV) was observed in patients with breast cancer who received reflexology treatments [9,10]. Foot reflexology, used concomitantly with conventional treatment, shows promise in decreasing the side effects induced by chemotherapy. In particular, it may decrease CINV, which is the side effect that concerns patients the most [11], as it decreases their overall quality of life [12,13] and induces metabolic complications [11]. In addition, CINV can lead to dose reduction, postponement of treatment, and even discontinuation of treatment [14], all of which can decrease the effectiveness of the treatment [15]. To control acute and delayed CINV, antiemetic drugs are prescribed; the main ones used are 5-hydroxytryptamine 3 receptor antagonists, dexamethasone, and neurokinin-1 inhibitor receptor antagonists [11,16,17]. But despite antiemetic therapy, CINV may persist [13]. In addition to the emetogenicity of the chemotherapy, various parameters may be associated, including risk factors (age, gender, alcohol use, history of motion sickness, and history of pregnancy-related vomiting) [12], treatment compliance [18] and the perceptual gap between health professionals and patients [19,20].

In 2018, in both sexes combined, lung cancer was the leading global cause of cancer death (18.4%), followed by digestive system cancer including colorectal (9.2%), stomach (8.2%), and liver (8.2%) [21]. According to national and international recommendations, adjuvant treatment for lung and digestive system cancer is platinum-based chemotherapy [22-26]. Anticancer therapy cisplatin has a high emetic risk (incidence of CINV >90%), while carboplatin and oxaliplatin have a moderate emetic risk (incidence of CINV is from 30% to 90%) [17].

### Objective

The aim of this study is to determine whether foot reflexology decreases the side effects induced by chemotherapy (specifically, CINV resulting from platinum-based chemotherapy) in patients with lung or digestive system cancer.

## Methods

### Trial Design

An open-label randomized controlled trial (RCT) will be carried out, in which patients are randomized to one of two groups at a 1:1 ratio: (1) conventional care with foot reflexology (40 patients) and (2) conventional care without foot reflexology (40 patients). Randomization is stratified on the type of cancer (digestive system or lung) and the presence or absence of metastases. The sponsor is the Hospices Civils de Lyon. The principal investigator is PJS. To design this trial, we used the CONSORT Statement for Randomized Trials of Nonpharmacologic Treatments [27].

### Eligibility Criteria of Participants

Eligibility requirements for this study are the following: patients aged ≥18 years; patients with lung cancer (eg, non–small cell lung carcinomas, small cell lung cancer, mesothelioma) or digestive system cancer (eg, colorectal cancer, pancreatic cancer, liver cancer); patients on platinum-based chemotherapy with or without radiation therapy concomitant (eg, digestive system cancer: FOLFOX, FOLFIRINOX, GEMOX, LV5FU2-CDDP; lung cancer: cisplatin-vinorelbine, pemetrexed-cisplatin, pemetrexed-carboplatin, carboplatin-paclitaxel); World Health Organization performance status ≤2; patients affiliated to the national social security system or equivalent; patients able to complete the questionnaires (comprehension of oral and written French language); and patients who provide written informed consent. The exclusion criteria are phlebitis; vena cava syndrome; weight loss >5% in the 3 months before inclusion date; uncontrolled pain; patients receiving morphine or morphine derivatives; brain metastases; patients receiving foot reflexology outside the study; patients under guardianship or curatorship or who have been deprived of their rights. All data will be collected from outpatients and inpatients.

### Description of Processes, Interventions, and Comparisons

The patients randomized to the intervention group will benefit from a foot reflexology session (30 minutes) at each chemotherapy session for four cycles. The reflexology sessions will be given during chemotherapy infusion every 2 or 3 weeks, depending on the chemotherapy protocol. The sessions will be administered by two qualified reflexologists (they were trained at the French school École des Techniques en Réflexologie).

In a meta-analysis study, Lee et al [28] determined that the optimal comparator is a control group with conventional care without foot reflexology or massage therapy.

Foot reflexology is CAM, based on the principle of acupressure, that helps the body restore homeostasis. It is a holistic approach that allows one to understand the body as a whole. Each part of the body is represented by a zone or reflex point on the foot. The reflexologist stimulates each reflex zone using specific thumb and finger techniques on the patient’s feet. Depending on the objective, the zones on the feet are stimulated using different types of pressure. During a session focused on the treatment of CINV, the reflexologist mainly stimulates the reflex points related to the digestive system, the lymphatic system, and kidneys to help the body eliminate toxins. The reflexology chart used in this clinical study is based on the one proposed by Eunice Ingham [29].

The reflex zones of the whole body are also found on the hands. During the first reflexology session, the reflexologist shows the patient the appropriate zones on the hands to relieve nausea. The patient will be able to stimulate these reflex points between each cycle if necessary (self-practice at home). Adherence of participants to reflexology is assessed with a diary. They have been instructed to use a diary every day between each cycle of chemotherapy to note episodes of nausea and vomiting. They also note if they took at least one antiemetic drug (on-demand treatment). If the patient uses self-massage, they note whether it has been effective.

The protocol of intervention was standardized by the reflexologists involved in this study. Over the course of the session, relaxation movements were incorporated after each stimulated reflex point. To calm nausea and vomiting, the reflexologist stimulated the upper and lower digestive reflex points, as well as smooth muscle reflex points (lymphatic system; kidneys and bladder; lungs; and thyroid and parathyroid). Encouraging deep relaxation to target anxiety involved the stimulation of the diencephalon reflex points, scapular belt reflex points, reflex points of the diaphragm, and reflex points of the spine.

### Enrolment, Screening, and Allocation

Participants will be enrolled by physicians at the Lyon Sud Hospital Centre thoracic and hepatogastroenterology departments. Eligible participants who wish to participate in the study are asked to sign a written informed consent form. The randomization procedure is performed by the Interactive Web Response System (IWRS) via ClinSight software (Ennov Clinical, version 7.5.710, Ennov Group). Participants are allocated to the intervention group (with foot reflexology) or to the control group (without foot reflexology) before starting their treatment. Clinical research assistants generate the random allocation sequence and assign participants to the intervention.

### Primary Objective

The primary objective is to assess the benefits of foot reflexology on CINV (from platinum-based chemotherapy) in patients with lung or digestive system cancer.

### Secondary Objectives

The secondary objectives are to assess the benefits of foot reflexology in terms of overall quality of life, anxiety, and self-esteem.

### Evaluation Criteria

#### Primary Outcome

The primary outcome is the relative change in the severity of nausea and vomiting, as assessed with a visual analogue scale (VAS). The patient is asked to mark their current nausea level on the horizontal line with anchor statements on the left (a happy face, no nausea=0 mm) and on the right (a very sick green face, paroxysm of nausea or vomiting=100 mm). Unlike vomiting, which is measurable by the number of episodes per day, nausea is a subjective experience. For that reason, we will use a VAS to evaluate the severity of nausea in patients [30]. For patients in the intervention group, this measurement will be done when the patient arrives at the outpatient or inpatient department and after the foot reflexology session, during the second cycle of chemotherapy. For patients in the control group, this measurement will be done when the patient arrives at the outpatient or inpatient department and before leaving the hospital, during the second cycle of chemotherapy. The assessment is performed by a nurse or clinical research assistant.

All patients will continue to receive standard antiemetic drugs (eg, 5-hydroxytryptamine 3 receptor antagonists, dexamethasone, and neurokinin-1 inhibitor receptor antagonists). According to chemotherapy protocols, oral antiemetic agents are included, and patients are instructed to complete the course.

#### Secondary Outcomes

##### Nausea and Vomiting

The benefits of foot reflexology on CINV will also be assessed with the diary completed every day by patients between the first and fourth cycle of chemotherapy. Every day, the patient assesses the frequency of nausea and vomiting, recording each episode of nausea and emesis, and assessing the intensity of nausea and vomiting when it is at its highest with a Likert scale (response modalities: “Very low,” “Low,” “Low Moderate,” “Severe,” “Very severe,” “Unbearable”).

##### Quality of Life

The benefits of foot reflexology on quality of life will be assessed by the relative change in the overall European Organization for Research and Treatment of Cancer Quality of Life Questionnaire (EORTC QLQ-C30) score [31]. The patient is asked to complete EORTC QLQ-C30 at the baseline and end of the study (fourth cycle of chemotherapy).

##### Anxiety

The benefits of foot reflexology on anxiety will be assessed by the relative change in the overall Hospital and Anxiety Depression Scale (HADS) score [32]. The patient is asked to complete HADS at the baseline and end of the study (fourth cycle of chemotherapy).

##### Self-Esteem

The benefits of foot reflexology on the level of self-esteem will be assessed at the end-of-study visit using the Body Image Questionnaire (BIQ) [33], which measures body image at a given time. The analysis of the BIQ takes into account the level of self-esteem assessed at the baseline using the Rosenberg scale [34].

#### Adverse Events

All adverse events are collected during this study and the assessment of causality with foot reflexology is at the physician's discretion.

### Sample Size

In a study reported by Billhult et al [35], the mean improvement for CINV (measured using a VAS) was 49.5% in the placebo group, and 73.5% in the massage group (with a common standard deviation of 32.2%). Assuming these same hypotheses, for a two-sided risk of 5%, it is necessary to include 40 patients per group to demonstrate a statistically significant difference between the two groups with a power of 90%.

### Type of Statistical Analysis Used

Study data are entered into a password-protected Excel spreadsheet (version 14.0, Microsoft Corp) accessible only to the project manager. Quantitative data will be described for the entire population, using the following descriptive statistics: the number, the number of missing values, the mean, the standard deviation, the median, the interquartile range, and the range. Qualitative variables will be summarized for the entire population using the following descriptive statistics: the frequency and percentage for each category of the variable and the missing values (missing values will be counted but are not included in the denominator of the calculation of frequencies).

For the primary endpoint, comparison of the relative change in the VAS between the two groups will be performed using a linear model adjusted on the type of cancer (digestive system or lung) and the presence of metastases (randomization stratification factors). This model allows the estimation of the difference in mean relative variation of VAS between the two groups, adjusted on potential confounding factors, with a 95% confidence interval. The VAS score may be transformed satisfy the assumptions of the linear model. If the assumptions of the model cannot be verified, the comparison between the two groups will be done using the van Elteren test. Specific estimates of the difference in relative VAS variation between the groups will be provided for both types of cancer, and by metastatic status.

The comparison of the frequency of episodes of CINV between chemotherapy cycles between the two groups will be performed using a mixed effects Poisson model, integrating the number of intercycle days as offset. The model will consider the intervention group and stratification criteria as fixed effects, and will incorporate one random intercept per patient.

The proportion of chemotherapy cycles in which the patient took at least one antiemetic drug will be compared between the two groups using a mixed effect logistic regression model. The model will consider the intervention arm and stratification criteria as fixed effects, and will incorporate one random intercept per patient.

The comparison of quality of life and anxiety between the two groups will be done using a Wilcoxon test.

The comparison of self-image between the two groups will be done using a linear model adjusted on the baseline self-esteem evaluated with the Rosenberg scale. A possible transformation of the BIQ score will performed to satisfy the assumptions of the linear model.

The analysis will be performed according to the intention-to-treat principle, considering all patients that completed the endpoint evaluation in the group that was allocated to them by the randomization. The time point for the primary endpoint has been defined to minimize the risk of missing data.

For the primary endpoint, a secondary analysis will be performed per protocol, including patients with the endpoint assessment, and for whom the strategy allocated during randomization was fully implemented (ie, patients allocated to the foot reflexology group that did not receive four sessions of foot reflexology will be excluded from the analysis).

### Regulatory and Ethical Considerations

This study was approved by the regional ethics committee (Île de France X CPP) on April 3, 2018 (No. ID RCB 2018-A00571-54). This study complies with the Reference Methodology (MR-001) developed by the French Data Protection Commission (Commission Nationale de l'Informatique et des Libertés), amended in October 2010, relating to the processing of personal data in clinical trials. This study follows the SPIRIT (Standard Protocol Items: Recommendations for Interventional Trials) guidelines [36].

Patients provide written informed consent that is dated and signed by the patient and the investigating physician. Patients in the control group will receive foot reflexology sessions at the end of their participation (two 30-minute sessions).

This study takes place in a university hospital. Each caregiver and investigator involved ensures optimal patient management. The project manager ensures communication and a close link between the caregivers, investigators, reflexologist, sponsor, and patients.

## Results

This study was approved by the regional ethics committee (Île de France X CPP) on April 3, 2018 (No. ID RCB 2018-A00571-54). Enrollment started in June 2018. Data analysis will be performed during the second quarter of 2020 and results will be published in the last quarter of 2020.

## Discussion

According to a European survey reported by Molassiotis et al [1], 35.9% of patients with cancer use CAMs, and after a diagnosis of cancer the use of CAMs increases by at least 30%. For varied reasons, some patients do not inform caregivers or health care professionals that they use CAMs [37,38]; however, certain CAMs could have potential interactions with conventional cancer treatments [39,40]. In parallel, oncologists lack adequate information about the safety and efficacy of CAMs to confidently inform their patients [41-43] and they have requested more rigorous evaluation [42,43]. Moreover, the World Health Organization’s Traditional Medicine Strategy emphasizes the importance of thorough evaluation; the objectives of this strategic approach are to inform policy; determine safety, efficacy, and quality; increase access; and promote the rational use of traditional medicine [44].

This RCT (Clinicaltrials.gov identifier: NCT03508180), which began in June 2018, assesses the benefits of foot reflexology. The expected results are a decrease in CINV and anxiety, as well as an improvement in quality of life and self-esteem. This will also allow the investigation of any potential difference in benefit between patients with lung cancer and patients with digestive system cancer, and patients with different stages of cancer (metastatic and nonmetastatic). The results will be available in the last quarter of 2020.

This study does have limitations. First, the results may not be representative of all cancers; patient recruitment was only done at one cancer center. Even if conventional treatments are similar within the various private and public health care centers in France, a larger multicenter study would ensure that the results are generalizable.

In conclusion, the current management of patients with cancer involves treatment with conventional medicine while offering supportive oncological care. If the results of this study are significant, foot reflexology may be a promising complement to conventional antiemetic drugs.

## Appendix

Multimedia Appendix 1 Ethical committee 04/03/2018.

## Abbreviations

BIQ: Body Image Questionnaire
CAMs: complementary and alternative medicines
EORTC QLQ-C30: European Organization for Research and Treatment of Cancer Quality of Life Questionnaire
HADS: Hospital and Anxiety Depression Scale
RCT: randomized controlled trial
SPIRIT: Standard Protocol Items: Recommendations for Interventional Trials
VAS: visual analogue scale
