# Supplementary material for: The Effects of Foot Reflexology on Chemotherapy-Induced Nausea and Vomiting in Patients with Digestive System or Lung Cancer: Protocol for a Randomized Controlled Trial
Source: JMIR Res Protoc. 2020 Jul 14;9(7):e17232. doi: 10.2196/17232 (PMC7388046; doi:10.2196/17232)
Supplement: Multimedia Appendix 1 [file resprot_v9i7e17232_app1.pdf]

HCL
HOSPICES CIVILS
DE LYON

ETHICAL APPROVAL

Study Title: ***Randomized study assessing the benefits of foot reflexology in patients with gastrointestinal or thoracic cancer with chemotherapy (REFYO-R Trial)*****.**

The Committee considered the information on this project at its meeting on April 03, 2018.

The Committee adopted the following deliberation for their review: FAVOURABLE OPINION.

Document validated by the Sponsor.

Done at Lyon on October 10, 2019.

Director of Clinical Research and Innovation Department

Lucilla MANSUY
Par Délégation, la Directrice de la Recherche
Clinique et de l'Innovation
